# The Effect of a Short Period of Supplementation with Glutamine Dipeptide on the Cognitive Responses after a Resistance Training Session of Women with HIV/AIDS: A Randomized Double-Blind Placebo-Controlled Crossover Study

**DOI:** 10.1155/2018/2525670

**Published:** 2018-04-03

**Authors:** Dayane Cristina de Souza, Junior Cesar da Silva, Felipe de Oliveira Matos, Alexandre Hideki Okano, Roberto Barbosa Bazotte, Ademar Avelar

**Affiliations:** ^1^Department of Physical Education, State University of Maringá, Maringá, PR, Brazil; ^2^Center of Mathematics, Computation and Cognition, Universidade Federal do ABC, São Bernardo do Campo, SP, Brazil; ^3^Department of Pharmacology and Therapeutics, State University of Maringá, Maringá, PR, Brazil

## Abstract

The aim of the present study was to investigate the effect of a short period of supplementation with glutamine dipeptide (GDP) on the acute responses to resistance training on the executive functions of people with HIV/AIDS. The sample consisted of 10 HIV+ women (45.00 ± 12.77 years old; 65.71 ± 12.04 kg; 1.54 ± 0.05 m) who were submitted to a randomized double-blind crossover procedure according to two experimental conditions: orally supplemented with 20 g/day of GDP or with maltodextrin for seven days. On the seventh day of supplementation all participants did cognitive function tests before and immediately after a resistance training session. Seven days of washout were adopted between conditions. Stroop and *N*-back tests were used to evaluate the executive functions. The training reduced the response time of each card in isolation and the latency time among them. GDP supplementation increased the magnitude of this effect, thus, reducing the latency time from the first to the last card in the Stroop test by almost 50% (*P* < 0.01). Considering the *N*-back test, there were no significant differences. It is suggested that GDP supplementation may increase the magnitude of the effect of an acute resistance training session in cognitive functions, particularly in the inhibitory control of people with HIV/AIDS. This trial is registered with NCT03236532.

## 1. Introduction

The immune system is not the only one to be compromised by the Human Immunodeficiency Virus (HIV) but it is possible to find such a virus in the Central Nervous System (CNS) [1]. It has been reported that the virus enters the CNS during the primary infection phase through a mechanism referred to as the “Trojan Horse,” in which the virus uses T-CD4+ and T-CD8+ lymphocytes, monocytes, and/or macrophages in order to cross the blood-brain barrier. This allows a virus replication in the CNS [2–4] and may trigger cognitive deficits of different intensities, with the dementia associated with HIV being the most serious of them [5].

The main regions of the brain affected by the virus are cortical and subcortical structures, such as frontal lobes [6] responsible for executive functions. These functions are understood as a set of cognitive abilities that control specific abilities, referring to the capacity of adaptive response to everyday situations, being the basis of many emotional and social competences [7].

The executive functions are comprised basically of three components, being (1) the working or operational memory, which is the capacity to integrate sensorial information in past experiences; (2) the preparatory set, which refers to the ability to organize and coordinate goal-directed behavior; (3) inhibitory control, which plays a role in the temporal organization of behavior and aims to suppress internal or external influences that may interfere with the sequence of ongoing action [7]. The impairment of these functions may significantly reflect the functioning of daily activities of HIV-infected persons [8]. Some studies also address the vulnerability of women in developing these neuropsychological impairments associated with HIV [9, 10]. Therefore, strategies to address impairments related to cognitive deficits in women living with HIV are needed.

Physical exercise programs and nutritional modulation have been investigated in isolation [11–13] and/or in combination [14] lately, in order to reduce or even prevent the progression of damage caused by different types of dementia in different populations. Specifically, the model of resistance training has been shown to be able to change the path of cognitive and motor decline, in addition to improving cerebral perfusion and executive functions [15–19], even after a single training session [20–22]. However, there are no similar studies on people with HIV/AIDS, although some studies show an association between active lifestyle and an improved brain function/integrity [23, 24]. In addition, practicing physical exercise may reduce the probability of neurocognitive impairment of people with HIV/AIDS by up to 50% [25]. Only one study investigated the effect of physical exercise intervention on the cognitive function, as their primary outcomes, and did not find significant results after 16 weeks of aerobic exercise [26] instigating the need for new research in this area with different exercise models on participants with HIV.

Regarding the nutritional aspects, the investigations that search to understand the mechanisms in the CNS that lead to the development of some dementia are highlighted. In this context, some authors have investigated the hypothesis of glutamine involvement in brain functions, making the key role of this amino acid in the CNS metabolism clear [27, 28].

Considering the HIV+ population, it has been shown that the serum glutamine concentration may reach a reduction of about 40%−50% due to the accelerated metabolism of the immune system cells [29, 30]. Therefore, glutamine supplementation may propitiate the increase of its concentration in the blood, enabling its action as a source of energy for the immune system. Furthermore, an increased availability of glutamine may favor the endogenous synthesis of glutathione [29], an important antioxidant that protects the cell, which has also been related to the treatment of cognitive disorders.

Based on the facts described above, this study aims at investigating the effect of a resistance training session associated with a short period of glutamine dipeptide (GDP) supplementation on the cognitive responses of women with HIV/AIDS, in view of the hypothesis that physical exercises may improve the executive functions in this population and that the GDP supplementation is able to increase this effect.

## 2. Methods

### 2.1. Participants

**Figure 1:**
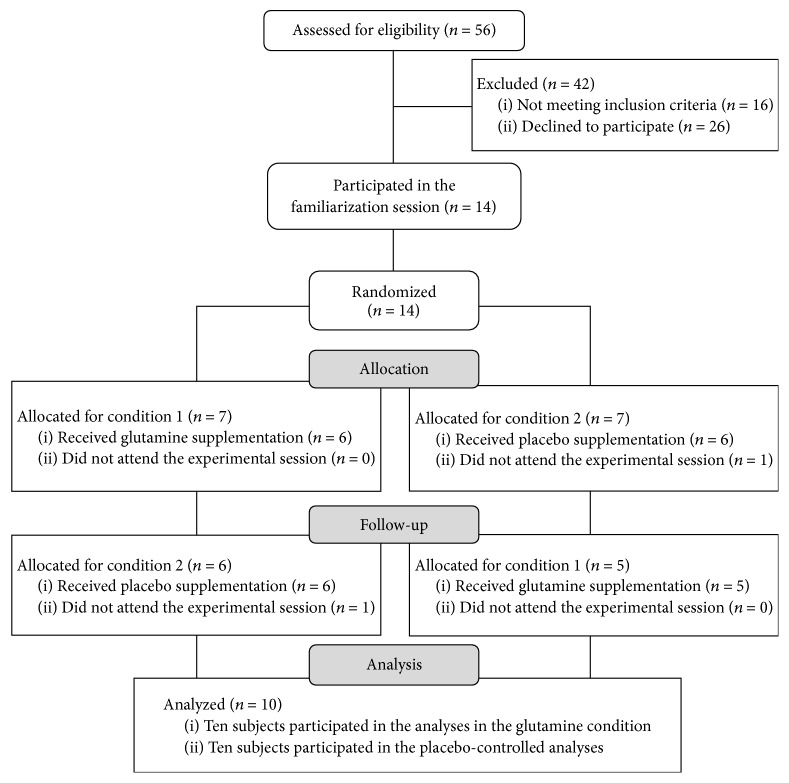
Flow diagram.

Initially 56 subjects were interviewed for eligibility in this study; of these 16 did not meet the inclusion criteria and 26 refused to participate. Fourteen women diagnosed with HIV+ participated in the randomization described in Figure 1.

As inclusion criteria, they should be over 18 years old; they should have regularly been using the Antiretroviral Therapy for more than six months; they should have a stabilized clinical picture and viral load; they should not have participated in physical training programs during the preceding six months; they should not show acute or chronic inflammations that could affect the practice of physical exercise; they should not have psychiatric disorders; and they should not be pregnant.

The individuals who met the inclusion criteria received clinical evaluation by the infectious physician responsible for the treatment based on the history of each patient, the laboratory tests, and the clinical picture.

The study was approved by the Standing Committee on Ethical Research with Humans of the State University of Maringá, PR, Brazil. The volunteers signed the Free Informed Consent Form, after being informed about the study proposal and the procedures they would be submitted to.

During the study period, four participants were not able to attend all the evaluations and were excluded from the final analysis. Therefore, 10 women participated in all the procedures and were part of the final sample of the study.

### 2.2. Experimental Design

**Figure 2:**
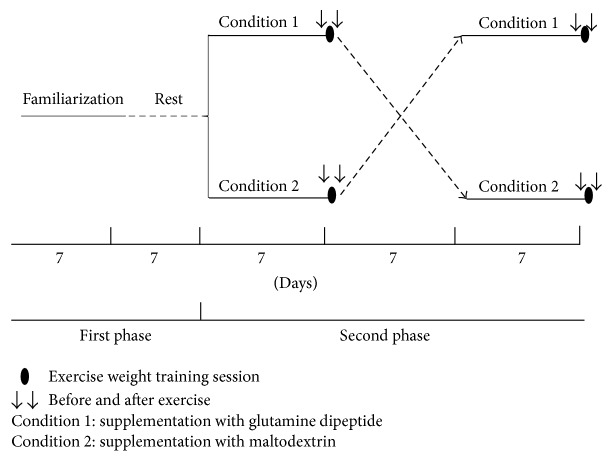
Scheme of the experimental design.

As described in Figure 2, the study had a total duration of five weeks divided into four phases. The first phase lasted two weeks and was characterized by familiarization with the exercises that would be performed in the second and fourth phases. This protocol consisted of four sessions of exercises, every 48 hours, which presented individuals to the same procedures that would be done in phases two and four. Four familiarization sessions were chosen, one of which aimed to learn the exercises without the insertion of loads. Then the participants could rest a week before starting the second phase.

In the second phase the participants were randomly separated in a double-blind manner to ingest GDP (Condition 1) or maltodextrin as a placebo (Condition 2) for seven days. On the seventh and last day of supplementation, they were submitted to the resistance training session. Before and immediately after the exercise session, cognitive tests were applied. The third phase consists of a rest period of one week. In the fourth phase the procedures performed in the second phase were repeated. However, the group that received GDP began to receive maltodextrin, and the group that received maltodextrin began to receive GDP, thus adopting a randomized double-blind crossover project.

### 2.3. Evaluation of the Cognitive Status

Considering the general evaluation of possible cognitive impairments, the Mini Mental State Examination (MMSE) was used, which provides information on different cognitive parameters, considering the level of education of the individual evaluated [31]. In order to assess the indicatives of HIV-associated dementia the International HIV Dementia Scale was applied [32].

### 2.4. Executive Functions

The Victoria Stroop color-word test was used to evaluate the executive functions [33], which consists of 72 stimuli, distributed in three cards with 24 items each (A, B, and C). The subject must verbalize the name of the colors with which the stimuli of each card were printed as quickly as possible; the time spent to read each of the cards separately was registered. The latency time, which is determined by the difference between the time spent with the responses to the three cards, was used to evaluate selective attention and inhibitory control.

The *N*-back test was used to evaluate the operational memory [34]. Visual stimuli were used in this study, which were represented by colors, divided into three levels (*N*1, *N*2, and *N*3) with 150 stimuli each. The score of each participant was obtained from the percentage values of the hits recorded in the three levels in a computerized version of the test.

### 2.5. The Resistance Training Protocol

The exercises were based on the Guideline for the prescription of exercise to be given to people with HIV/AIDS [35]. The training session consisted of seven exercises with weights (chest press, leg press 45°, lat pulldown machine, knee extension, triceps pulley, knee curl, and Scott biceps curl machine) involving different muscle groups with three series per exercise.

The recovery interval adopted was of 90 seconds between the series and 120 seconds between exercises. The number of repetitions used in each one of these series was of 8–12 repetitions by applying the method of fixed loads. The loads were compatible with the number of repetitions stipulated for each exercise. The load determination occurred during familiarization sessions.

The OMNI Resistance Exercise Scale (OMNI-RES) of effort subjective perception was used to determine and control the loads of each exercise. The loads used during the experimental sessions corresponded to the intensity equivalent to the interval from five to seven (5–7) of the OMNI-RES scale.

In order to achieve such a purpose, prior to the beginning of familiarization with the exercises, the OMNI-RES scale anchoring was performed. This procedure consists of placing the patient in the smallest and highest possible load in each exercise so that he/she can differentiate a smaller and a greater subjective perception of effort.

### 2.6. Supplementation Protocol

The participants were randomly double-blinded indicated to receive either GDP (20 g/day) or maltodextrin (20 g/day) according to the literature [29]. The substances were packed in sachets containing GDP or maltodextrin (placebo). The packages were identical and the substances used had a similar color and texture. Participants were instructed to dilute GDP or maltodextrin in 300 ml of water and ingest it after lunch. In case of forgetfulness, they were suggested to ingest it soon after dinner. All participants were instructed to maintain their routine eating habits throughout the duration of the study.

### 2.7. Statistical Analysis

The descriptive statistics were used to characterize the sample. Shapiro-Wilk test was used for data distribution analysis. For the variables that showed a parametric distribution, the independent *t*-test was used in order to compare the preexercise values. For the variables that did not show a parametric distribution, Mann–Whitney *U* test was used with the same purpose. Mauchly's sphericity test was used to verify the sphericity of the data. Considering the variables in which the sphericity was violated, the analyses were adjusted by using the Greenhouse-Geisser correction. The two-way ANOVA (time × supplement) for repeated measurements was used for intra- and intercondition comparisons. Bonferroni post hoc test was used when a significant *F* ratio was identified either for an isolated effect of the factors analyzed or for the interaction among them. All data were treated with the Statistics software, version 7.0. The significance level of *P* < 0.05 was adopted for all analyses.

## 3. Results

The data related to the characterization of the participants showed in Table 1 include the information obtained in the questionnaires applied to detect the presence of cognitive deficit. The results show that, in spite of being infected by the virus, the participants do not have cognitive impairments of the domains evaluated by the International HIV Dementia Scale and the MMSE.

The results obtained by using Stroop test are shown in Table 2. The data are presented by means of the pre- and postexercise time for each of the cards (A, B, and C) individually.

Stroop test is considered a neuropsychological test sensitive to impairment in the executive functions, more specifically attention and inhibition. The results showed an isolated effect of the time factor (training session) considering the independent analysis of each card (A, B, and C) with a significant reduction in the comparisons between before and after acute resistance training sessions, in addition to a reduction in the latency time from card B to card C (*P* < 0.01) and from card A to card C (*P* < 0.01).

The interaction from time versus supplementation in the latency time from the first to the last card showed that the participants, after being supplemented for seven days with GDP, presented a significant decrease (*P* < 0.01) in the response time between the cards, when compared to the pre- and postexercise evaluation.

For the central executive component of the operational memory, measured by visual stimuli and using the *N*-back test, no effect was found (GDP: before 34.7  ± 19.0 and after 43.3  ± 16.4; placebo: before 39.8  ± 18.7 and after 39.2  ± 16.4), both in an isolated way (*P* time = 0.29; *P* supplement = 0.95) and with the interaction between them (*P* = 0.23).

## 4. Discussion

The findings of the present study indicate that resistance training can improve the cognitive responses of people with HIV/AIDS, thus intervening both in selective attention and in inhibitory control, which refer to one of the three central components of executive functions [7], that is, the capacity of focusing attention on specific stimuli and disregarding irrelevant stimuli [36].

The favorable effect of resistance training in the sense of reducing the cognitive and motor decline, besides improving cerebral perfusion and executive functions [15, 16, 19], has been reported in previous studies [20–22]. However, these results were obtained from people of different ages [20–22, 37–41] and pathologies [37, 42, 43] in addition to the absence of studies that evaluate the effect of the training on the cognition of HIV-infected individuals.

It is worth emphasizing that HIV may lead to important changes in cognition [44] and, therefore, it is possible that it also directly influences the responses to exercise. Thus, the results of the present study may be a reference base when dealing with the effect of this training model on the cognition of people with HIV/AIDS.

In the literature, the neuroprotective effect of physical exercise considering the decline of cognitive functions and the risk of dementia, such as Alzheimer's disease, has been pointed out [42, 43]. Therefore, the results obtained suggest that, as in the elderly population, resistance training, even acutely, may also positively influence cognitive aspects in the HIV+ population.

The cognitive improvement by doing physical exercise would be related to the elevated release of neurotransmitters (norepinephrine, serotonin, and endorphin) and their precursors [45] as well as an increased blood flow in different areas of the brain, representing a higher supply of oxygen and nutrients and, consequently, a higher energy supply [46].

The hypothesis of this study that GDP supplementation could increase the effect of the exercise on the executive functions was confirmed in the interaction analysis of the time versus supplement and in the latency time from the first card (A) to the last one (C) by using Stroop test. The participants, when supplemented with GDP, showed a mean reduction of 47.1% in the response time among the cards, whereas under the placebo condition they showed a mean reduction of only 10.5%.

Considering that people with HIV/AIDS have a reduced serum concentration of glutamine [29, 30, 47, 48], it is possible that the main performance of this amino acid in the cognitive aspects is related to the increase of neurotransmitter amino acid synthesis, although it is not clear if this occurs through the excitatory or inhibitory pathway, thus requiring further studies to confirm this premise.

Another possibility is related to the role of glutamine as a source of energy for the brain [44], in addition to the increase of the endogenous synthesis of glutathione [29]. People who live with HIV have reduced the glutathione levels, which is similar to the impacts caused in the CNS through some processes, that is, aging and different diseases, such as Alzheimer's and Parkinson's [29, 49]. Glutathione is an antioxidant used in the combat of cognitive disorders, since it acts as an important neuromodulator in the CNS [47, 50].

Besides being a precursor of glutathione, the glutamine plays the same role in relation to glutamate, the main excitatory CNS neurotransmitter, aspartate, and gamma-aminobutyric acid (GABA), the main inhibitory neurotransmitter [48].

Considering that high levels of glutamate can be toxic to the CNS [51], the findings by Borges-Santos et al. [29] show a positive response, which evidences that glutamine supplementation is able to significantly increase serum concentrations of glutathione in people with HIV, without significantly increasing the glutamate concentrations.

However, in order to confirm these possible associations, further studies should be carried out with more accurate methods of evaluation and able to determine if the GDP supplementation may increase the concentrations of glutamine and its precursors in the CNS.

Despite the evidence of the glutamine role in the CNS, its involvement in cognitive responses remains unclear. This is because some of the studies that investigated the possible effects of nutritional modulation on cognitive responses used vitamin and/or lipid supplements and the results do not make it clear if there really is any ergogenic effect.

When analyzing the nutrition and cognition relation, it is seen that the resistance training associated with protein supplementation would improve the speed of information processing in the elderly. The resistance training itself has improved attention and working memory. The few studies that evaluated the impact of glutamine supplementation on cognitive responses found improvement in children's learning index [13] and in the reaction time in endurance athletes [52]. However, in the present study no difference was found in the comparison of prevalues (glutamine versus placebo), which shows that one week of glutamine supplementation alone did not result in cognitive improvement.

In tasks as the *N*-back, the content of the operational memory needs to be constantly manipulated, since the target stimulus is updated to each item shown [53]. In this study, no significant change was found in the assessment of operational memory, assessed by the *N*-back test.

Knowing that the operational memory is a system of limited capacity, it is common that the individuals evaluated have a poor performance as the test level increases [54]. It was expected that, after the resistance training session and/or with the GDP supplementation, this performance would improve; however this did not happen, both in the analysis of the isolated factors and in the analysis of the interaction between these two factors.

It is important to highlight that the supplementation time and the sample size may have influenced the results of the present investigation and may have eased the possible impact of the association between resistance training and GDP supplementation considering the parameters evaluated. Another relevant aspect is the lack of a more sensitive evaluation method to detect possible changes in a short period of time.

Despite these limitations, the results obtained are promising, in particular the demonstration of the positive impact of physical exercise on the cognitive responses of people with HIV/AIDS. In addition, the results raise the possibility of an additional benefit from GDP supplementation for a short period of time. Therefore, further studies should be carried out with the aim of evaluating the chronic impact of the exercises and/or GDP supplementation on the cognitive responses of people with HIV/AIDS.

## 5. Conclusion

Acute resistance training was sufficient to improve in the executive function, specifically in the inhibitory control in women with HIV/AIDS. This response was even more expressive when associated with the GDP supplementation, but there were no significant changes in the operational memory.

## Figures and Tables

**Table 1:** Characterization of the participants of this study (*n* = 10).

Variable	Mean	Standard deviation
Age (years)	45.0	±12.8
BMI (kg/m^2^)	27.4	±4.0
CD4 (mm^3^)	369.7	±202.0
CD8 (mm^3^)	802.8	±308.7
CD4/CD8	0.4	±0.3
ART time (years)	6.1	±5.6
MMSE (score)	27.5	±1.0
IHDS (score)	11.6	±0.5

BMI = Body Mass Index; ART = Antiretroviral Therapy; MMSE = Mini Mental State Examination; IHDS = International HIV Dementia Scale.

**Table 2:** Stroop test results when comparing maltodextrin (placebo) versus glutamine dipeptide (GDP) supplementation before and after an acute resistance training session (*n* = 10). Values expressed as mean ± and standard deviation.

	Placebo	GDP	Effects	*F*	*P*
*T*_latency: A-B (seconds)			ANOVA		
Pre	1.6 ± 3.7	2.3 ± 3.2	Supplement	0.01	0.93
Post	2.6 ± 2.0	2.1 ± 2.1	Time	0.34	0.57
			Supplement × time	0.97	0.34
*T*_latency: B-C (seconds)			ANOVA		
Pre	7.0 ± 7.1	7.9 ± 7.0	Supplement	0.05	0.83
Post	5.2 ± 4.7	3.3 ± 3.1	Time	11.60	<0.01
			Supplement × time	0.97	0.34
*T*_latency: A-C (seconds)			ANOVA		
Pre	8.6 ± 7.5	10.2 ± 6.2	Supplement	0.03	0.87
Post	7.7 ± 5.3	5.4 ± 3.6^*∗*^	Time	13.44	<0.01
			Supplement × time	6.28	0.02
Card: A (seconds)			ANOVA		
Pre	15.2 ± 3.6	15.2 ± 1.9	Supplement	0.49	0.49
Post	13.2 ± 2.0	14.5 ± 1.8	Time	8.02	0.01
			Supplement × time	1.41	0.25
Card: B (seconds)			ANOVA		
Pre	16.8 ± 3.8	17.6 ± 3.7	Supplement	0.29	0.60
Post	15.6 ± 3.3	16.5 ± 2.8	Time	7.18	0.01
			Supplement × time	0.01	0.98
Card: C (seconds)			ANOVA		
Pre	23.8 ± 9.4	25.5 ± 7.5	Supplement	0.01	0.93
Post	20.9 ± 6.4	19.8 ± 4.9	Time	25.59	<0.01
			Supplement × time	2.66	0.12

^*∗*^ *P* < 0,01 versus Pre; *T*_latency = Time of Latency.
